# An electron paramagnetic resonance time‐course study of oxidative stress in the plasma of electronic cigarette exposed rats

**DOI:** 10.1113/EP092064

**Published:** 2024-08-01

**Authors:** Murugesan Velayutham, Amber Mills, Valery V. Khramtsov, I. Mark Olfert

**Affiliations:** ^1^ In vivo Multifunctional Magnetic Resonance Center West Virginia University School of Medicine Morgantown West Virginia USA; ^2^ Center for Inhalation Toxicology West Virginia University School of Medicine Morgantown West Virginia USA; ^3^ Department of Biochemistry and Molecular Medicine West Virginia University School of Medicine Morgantown West Virginia USA; ^4^ Department of Physiology, Pharmacology & Toxicology West Virginia University School of Medicine Morgantown West Virginia USA; ^5^ Department of Human Performance, Division of Exercise Physiology West Virginia University School of Medicine Morgantown West Virginia USA

**Keywords:** electron paramagnetic resonance, 1‐hydroxy‐3‐carboxymethyl‐2,2,5,5‐tetramethyl‐pyrrolidine, inhalation exposure, vaping, vascular dysfunction

## Abstract

**Abstract:**

The long‐term consequences of electronic cigarette (Ecig) use in humans are not yet known, but it is known that Ecig aerosols contain many toxic compounds of concern. We have recently shown that Ecig exposure impairs middle cerebral artery (MCA) endothelial function and that it takes 3 days for MCA reactivity to return to normal. However, the sources contributing to impairment of the endothelium were not investigated. We hypothesized that the increased levels of oxidative stress markers in the blood are correlated with impaired MCA reactivity. We used electron paramagnetic resonance (EPR) spectroscopy to examine plasma from 4‐month‐old male Sprague–Dawley rats that were exposed to either air (*n* = 5) or 1 h Ecig exposure, after which blood samples were collected at varying times after exposure (i.e., 1–4, 24, 48 and 72 h postexposure, *n* = 4 or 5 in each time group). The EPR analyses were performed using the redox‐sensitive hydroxylamine spin probe 1‐hydroxy‐3‐carboxymethyl‐2,2,5,5‐tetramethyl‐pyrrolidine (CMH) to measure the level of reactive oxidant species in the plasma samples. We found that EPR signal intensity from the CM^•^ radical was significantly increased in plasma at 1‐4, 24 and 48 h (*P *< 0.05, respectively) and returned to control (air) levels by 72 h. When evaluating the EPR results with MCA reactivity, we found a significant negative correlation (Pearson's *P *= 0.0027). These data indicate that impaired cerebrovascular reactivity resulting from vaping is associated with the oxidative stress level (measured by EPR from plasma) and indicate that a single 1 h vaping session can negatively influence vascular health for up to 3 days after vaping.

**Highlights:**

- **What is the central question of this study?**
  Does the time course of oxidative stress triggered by electronic cigarette exposure follow the cerebral vascular dysfunction?
- **What is the main finding and its importance?**
  Electron paramagnetic resonance analysis shows that the oxidative stress induced after a single 1 h exposure to electronic cigarette aerosol takes ≤72 h to return to normal, which mirrors the time course for vascular dysfunction in the middle cerebral artery that we have reported previously.

## 1 INTRODUCTION

Electronic cigarettes (Ecigs) have been distributed on the global market both as a safe alternative to combustion cigarettes and as a potential approach to aid smoking cessation. Although the long‐term health consequences of Ecig use in humans are not yet fully known owing to the relative novelty of this product, the available studies from Ecig exposure in humans and animals show remarkedly similar findings in the context of cardiovascular risk and concerns. For example, vaping leads to increased arterial stiffness in humans (Buchanan et al., 2020; Larue et al., 2021; Meng et al., 2022; Skotsimara et al., 2020) and animals (Olfert et al., 2018; Szostak et al., 2020). Endothelial vascular dysfunction is also observed in humans (Belkin et al., 2023; Carnevale et al., 2016; Chaumont et al., 2018) and animals (Jin et al., 2021; Mills et al., 2022, 2023; Rao et al., 2022) with Ecig exposure. Likewise, increased risk for myocardial infarct (MI), decreased heart function and/or increased arrhythmias have been reported with Ecig use in humans (Alzahrani et al., 2018; Ashraf et al., 2023; Wang et al., 2018) and animals (Carll et al., 2022; Dai et al., 2023; Qiu et al., 2023). Although not all reports in humans show a greater risk of MI (Berlowitz et al., 2022; Farsalinos et al., 2019), there is a growing consensus of data emerging from acute animal and human studies that cannot be ignored (Rose et al., 2023).

We have recently shown that Ecig exposure impairs endothelium‐dependent vasodilatation via a nitric oxide (NO)‐dependent pathway in the middle cerebral artery (MCA) of rats (Mills et al., 2022) and mice (Mills et al., 2023). The important finding from the rat study (Mills et al., 2022) was that the impaired MCA reactivity persisted for ≤72 h (3 days) after a single 1 h exposure, and the effect was similar whether rats were exposed to 20 or 60 puffs. At the time, the potential sources contributing to impairment of the endothelium were not fully investigated concerning oxidative stress (OS). In the present study, we used electron paramagnetic resonance (EPR) spectroscopy to test the hypothesis that the time course of OS in the plasma is correlated with the manifestation and recovery from vascular impairment that we observed in the MCA, and thus provide strong evidence that OS is a primary contributor to Ecig‐induced cerebral vascular dysfunction.

## 2 MATERIALS AND METHODS

### 2.1 Ethical approval

All procedures were conducted with approval from West Virginia University Animal Care and Use Committee (#16‐05003053) and conformed to the previously described principles and regulations for animal experimentation. All surgical procedures were performed under deep general anaesthesia (inhaled isoflurane) to minimize pain or discomfort and were terminal (owing to thoracotomy and exsanguination from blood collection).

### 2.2 Study design and exposure

Sprague–Dawley rats (4 months old) were exposed to either air (*n* = 5) or a single 1 h Ecig aerosol exposure followed by euthanasia and organ/tissue collection at different time points postexposure (*n* = 4–5 per time point), corresponding to 1–4, 24–28, 48–52 and 72–76 h after exposure [i.e., noted as day (D)0, D1, D2 and D3 postexposure for simplicity]. The collected blood was centrifuged at 1600 *g* for 10 min at 4°C. After centrifugation, the plasma was aliquoted in microfuge tubes, immediately flash‐frozen in liquid nitrogen and stored at −80°C until used for analysis.

The Ecig exposure conditions and rationale have been described in full previously (Mills et al., 2022). In brief, whole‐body exposure to Ecig aerosol was performed using a Joyetech Ecig device with an 0.5 Ω atomizer set at 17.5 W, with e‐liquid containing 50:50 (v:v) of vegetable glycerin (Avantor J.T. Baker #2143‐01) and propylene glycol (Fisher #P355‐1) without nicotine or flavouring.

### 2.3 Electron paramagnetic resonance spectroscopy

At the time of processing, frozen plasma samples were thawed on ice, spun at 1500 r.p.m. for 5 min, and 100 µL was incubated with the EPR spin probe 1‐hydroxy‐3‐carboxymethyl‐2,2,5,5‐tetramethyl‐pyrrolidine (CMH; 0.2 mM) for 30 min at 37°C. The reactive oxidant species (ROS) in the plasma oxidize CMH to produce a stable EPR‐active CM^•^ radical (Majumder et al., 2022). Thereafter, samples were flash‐frozen in liquid nitrogen and stored at −80°C until being used with an EPR spectrometer (Bruker ELEXSYS E580 spectrometer) operating at X‐band with 100 kHz modulation frequency.

At the time of EPR measurements, frozen samples were rapidly thawed to room temperature and immediately loaded (50 µL) into glass capillary tubes, sealed at one end using Critoseal clay, then placed inside an EPR quartz tube (4 mm in outer diameter).The quartz tube was placed inside the resonator/cavity, and spectra were recorded at room temperature. The EPR instrument settings were as follows: microwave frequency, 9.854 GHz; sweep width, 100 G; microwave power, 23.77 mW; modulation amplitude, 1 G; modulation frequency, 100 kHz; conversion time, 29.3 ms; sweep time, 30 s; and number of scans, 1. Acquisition of EPR data was performed using Bruker Xepr software. The signal intensity was generated using the first peak (low‐field) height of the spectrum.

### 2.4 Statistical analyses

Data are presented as the mean ± SD. Data were evaluated for normality using the Kolmogorov–Smirnov test. One‐way ANOVA was used to evaluate exposure group differences. Dunnett's *post hoc* test was used to determine differences between groups. Pearson's correlation was used to determine the relationship between the plasma EPR signal and the maximal vasodilatation observed for the MCA from each animal. Significance was accepted if *P* ≤ 0.05. All data were analysed using GraphPad Prism v.10.2.3 software.

## 3 RESULTS

**FIGURE 1:**
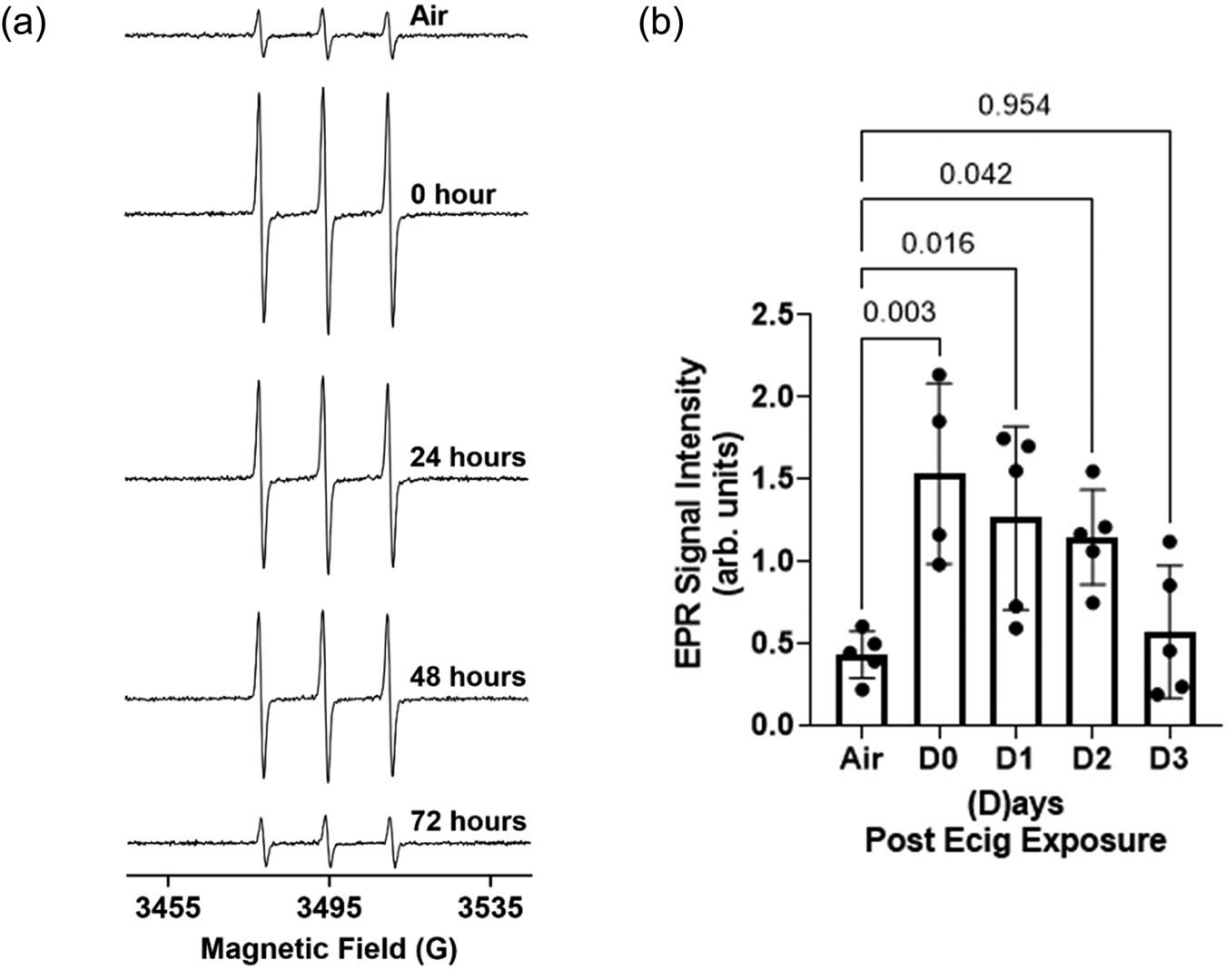
Electron paramagnetic resonance studies of oxidation of CMH by rat plasma samples. The reactive oxidant species in the plasma oxidize CMH to produce a stable EPR‐active CM^•^ radical. (a) Representative room temperature X‐band EPR spectra of CM^•^ radical in the rat plasma samples. Rat plasma samples were obtained at 0, 24, 48 or 72 h (i.e. D0, D1, D2 and D3, respectively) after rats were exposed to Ecig. (b) Summary and plot of EPR signal intensity of CM^•^ radical in the rat plasma samples. Abbreviations: CMH, 1‐hydroxy‐3‐carboxymethyl‐2,2,5,5‐tetramethyl‐pyrrolidine; EPR, electron paramagnetic resonance.

**FIGURE 2:**
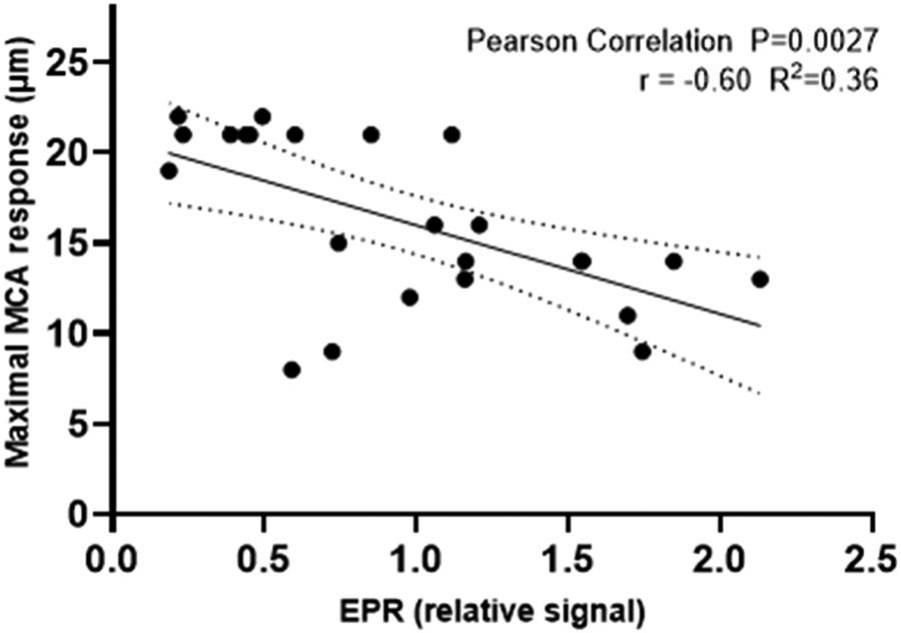
Scatter plot showing significant negative correlation between maximal MCA reactvity to acetylcholine (10^−4^ M) and EPR single intensity, indicating that the increasing EPR signal (i.e., increased oxidative stress) is associated with worsening MCA reactivity. Abbreviations: EPR, electron paramagnetic resonance; MCA, middle cerebral artery.

Representative EPR spectra of the plasma samples are shown in Figure 1a. The signal intensity of the first peak of the EPR spectra was used for quantification of the level of OS. The measurements show (compared with air controls) that the highest level of OS occurs within 1–4 h after Ecig exposure (i.e., D0, *P* = 0.003) and begins to decrease by ∼24 h (D1, *P* = 0.016) and 48 h (D2, *P* = 0.042) (Figure 1b). By 72 h (D3, *P* = 0.954), the EPR signal has returned to control levels. Shown in Figure 2, there is a significant negative correlation between the EPR signal intensity and the maximal MCA reactivity response to 10^−4^ M acetylcholine‐induced vasodilatation. The MCA responses were reported previously by Mills et al. (2022).

## 4 DISCUSSION

We are not the first to report or find that Ecig exposure increases OS, but to our knowledge, these are the first data clearly to show a robust time‐course relationship between OS (in plasma) and the development and recovery from vascular impairment. Given the time that OS remains elevated (and the accompanying vascular dysfunction) after a single exposure, these data suggest that EPR spectroscopy might be a useful non‐invasive technique to evaluate exposure to Ecigs.

Free radical oxidants are often short‐lived compounds and therefore can be difficult to assess fully. Although many studies (using a wide range of techniques) have observed increased OS/ROS in association with Ecigs (see reviews: Ding et al., 2023; Tarran et al., 2021), most studies assessing biological samples have evaluated ROS at only a single time point after cigarette or Ecig use. Although data from a single time point are useful, free radical production and redox balance are highly dynamic, thus it often remains unknown (unless multiple time points are included) whether the time point selected/measured represents the peak or some immediate response. Our data are able to establish that there is a significant inverse relationship between OS and MCA reactivity. Although this association alone cannot establish a causal relationship between ROS and vascular dysfunction, we have reported in our previous publication that MCA dysfunction in these animals is restored to normal when TEMPOL (a superoxide dismutase mimetic) is included in the *ex vivo* arteriography preparation (Mills et al., 2022). Thus, the outcome seen here (and when combined with the existing literature) provides compelling evidence that OS/ROS are principal contributors to the cerebrovascular dysfunction induced by Ecig exposure.

A unique aspect of this study is that, to our knowledge, this is the first report to show EPR analyses from biological tissue/liquid (i.e., plasma) to assess OS in response to Ecig exposure. Many studies have used EPR to assess the ROS production in the aerosol cloud produced by Ecigs (Bitzer et al., 2018, 2020; Goel et al., 2015; Lerner et al., 2015). These data establish that Ecig aerosol, like cigarette smoke, produces ROS that are inhaled into the lungs. These studies have also found that the level of free radical production can be influenced by the presence of flavours (Bitzer et al., 2018; Goel et al., 2015; Lerner et al., 2015), but can also be produced solely from the base solution (vegetable glycerin and propylene glycol) (Goel et al., 2015). Furthermore, ROS levels might also be dependent on the heating temperature, the age of the coil and the type of coil material (Cirillo et al., 2019; Tran et al., 2023). It is interesting to note that the role of nicotine is less clear, because one study demonstrated that only the flavouring agent, and not nicotine, sets the redox status for endothelial cells in culture (Kerasioti et al., 2020). Although understanding the level of ROS in the aerosol is important, it still leaves the question of whether ROS are present in the tissue or blood after vaping. Here, we show that they are, and that the time course of ROS closely matches our data showing vascular impairment with a single exposure to Ecigs (Mills et al., 2022). It should be noted that we used a non‐specific EPR spin probe (i.e., CMH) to assess the level of OS, and plasma can contain several oxidizing species, such as peroxynitrite, NO, superoxide radical, hydrogen peroxide and transition metals, which contribute to the one‐electron oxidation of CMH to CM^•^ radical. Although the probe we used cannot identify specific ROS, the advantage of the EPR technique is that there are different spin‐traps that can uniquely identify ROS, and EPR approaches have no contribution from the background signals to the results. Thus, detection of ROS via EPR is a powerful tool that can provide robust evaluations of OS.

There are some technical limitations in the present study. First, we have a small sample size, but even with the small number of animals, the correlation between EPR signal and MCA reactivity (which is based on paired variables) adds confidence to our interpretation. Second, the use of whole blood (rather than plasma) immediately introduced to CMH would have been more ideal for our EPR analyses: (1) rapidly to drive formation of the stable CM^•^ adduct; and (2) to include the influence of red blood cells or immune cells. Instead, our plasma samples were flash‐frozen and underwent one freeze–thaw cycle before addition of CMH, hence we cannot rule out the possibility that our EPR signal might have been attenuated. However, given that all the samples were processed in the same manner, we would argue that any effect would have influenced all the samples and, as such, should not prevent or interfere with the interpretation of results between the groups. Notably, CM^•^ adducts stored at −80°C are stable for >6 months (Berg et al., 2014).

## 5 PERSPECTIVES

Our data were obtained from an animal model, hence caution is warranted in extrapolating to human end points. But it is noteworthy that our exposure protocol (i.e., using a single 1 h session consisting of 20–60 puffs) is likely to underestimate use by humans. For example, people are reported to take 20–36 puffs per vaping session and often have multiple (e.g., 5–15) sessions each day (Addicott et al., 2023; Li et al., 2021), resulting in a wide range of daily exposure (e.g., between 100 and >500 total number of puffs/day) (Li et al., 2021; Wagener et al., 2021). In humans, 10–16 puffs on an Ecig are reported to reduce cerebrovascular reactivity within the same day as the exposure (Ben Taleb et al., 2023; Campbell et al., 2022; Caporale et al., 2019). Most reports of circulating ROS after Ecig exposure that we found in humans are also limited to same‐day analyses (Carnevale et al., 2016; Chatterjee et al., 2019; Kelesidis et al., 2020); therefore, to our knowledge, it is not established whether the same effect persists for 48–72 h in humans, as we see in animals.

The temporal relationship between OS and vascular dysfunction seen here (after one Ecig exposure bout) provides an important perspective into the potential development of chronic disease with long‐term Ecig use. If the acute effects of vaping on vascular impairment last for 48–72 h postexposure, then individuals who vape daily (or even only once every 3 days) might maintain a consistent state of elevated OS, leading to altered endothelial cell homeostasis. If the same time course holds true for humans, we speculate that the potential risk for cardiovascular and/or cerebrovascular disease is likely to be greater in people who use Ecigs more than once every 3 days compared with those who vape less often (i.e., >3 days between exposure bouts).

## AUTHOR CONTRIBUTIONS

Murugesan Velayutham, Amber Mills and I. Mark Olfert conceived and designed the study; Murugesan Velayutham and Amber Mills collected data and/or performed experiments; Murugesan Velayutham, Amber Mills, Valery V. Khramtsov and I. Mark Olfert analysed data and interpreted results of the experiment; Murugesan Velayutham and I. Mark Olfert prepared figures and drafted the manuscript; all authors helped to edit, revise and approve the final version of the manuscript. All authors agree to be accountable for all aspects of the work in ensuring that questions related to the accuracy or integrity of any part of the work are appropriately investigated and resolved. All persons designated as authors qualify for authorship, and all those who qualify for authorship are listed.

## CONFLICT OF INTEREST

None declared.

## Data Availability

All the data that support the findings of this study are presented in Figures 1 and 2.
